# From Theater to Clinic: A Retrospective Evaluation of Local Anesthetic Transperineal Biopsy for the Rapid Diagnosis of Prostate Cancer

**DOI:** 10.7759/cureus.92204

**Published:** 2025-09-13

**Authors:** James A Temple, Shahzad Ahmad

**Affiliations:** 1 Urology, Epsom and St Helier University Hospitals NHS Trust, London, GBR

**Keywords:** local anesthetic, prostate biopsy, prostate cancer diagnosis, trus/tp la prostate biopsy, urology clinic

## Abstract

Background

Prostate cancer is a common malignancy among men, and health systems are adopting faster diagnostic pathways to improve outcomes. Traditional transrectal ultrasound-guided (TRUS) biopsies under sedation carry a notable risk of infection, prompting a shift toward transperineal (TP) prostate biopsy under local anesthesia (LATP) in outpatient settings. We aimed to evaluate a new clinic-based LATP pathway for prostate cancer diagnosis in terms of speed, safety, and patient experience.

Methods

We retrospectively reviewed all patients referred with suspected prostate cancer who underwent the outpatient LATP pathway at a London NHS trust over a six-month period (May to November 2024). The pathway included an initial one-stop clinic visit (assessment and prostate-specific antigen test), rapid multiparametric MRI, and an office-based TP prostate biopsy under local anesthesia. Data were collected from electronic records and a post-biopsy patient survey. Key outcomes included diagnostic timelines (from urgent referral to biopsy to definitive diagnosis), compliance with the 28-day Faster Diagnosis Standard (FDS), procedure-related complications within 30 days, and patient-reported measures (pain, tolerability, satisfaction, and anesthesia preference).

Results

A total of 115 men (mean age ~68 years) were included. The mean interval from urgent referral to biopsy was ~10 days, and from biopsy to confirmed diagnosis ~5 days, resulting in an average referral-to-diagnosis time of about 14.5 days. Overall, 85.4% of patients received a definitive diagnosis within 28 days of referral, exceeding the national FDS target of 77%. Only 14.6% exceeded 28 days, typically by just a few days due to minor delays. Prostate cancer was diagnosed in 65% of patients, of whom 76% had clinically significant disease (Grade Group ≥2). Complications were infrequent and minor. There were 2 cases of urosepsis (1.7%), both managed successfully with brief hospitalization, and 2 vasovagal episodes (1.7%) with no lasting effects. No patients experienced urinary retention, significant bleeding, or other serious adverse events (overall complication rate ~3.5%). Patient feedback was positive: the average pain score during biopsy was 6.2/10 (moderate discomfort), but tolerability was rated favorably (mean 3.2/10 on a 0-10 difficulty scale). The mean satisfaction score with the diagnostic process was 8.2/10. Among surveyed patients, 68% indicated they would choose the same local anesthetic biopsy again, 20% were unsure, and 12% would prefer sedation or general anesthesia if another biopsy were needed. Patients valued avoiding a hospital stay and receiving results quickly.

Conclusions

Implementation of an outpatient LATP biopsy pathway substantially accelerated prostate cancer diagnosis, with most patients receiving a diagnosis within two weeks of referral and more than 85% within 28 days. This fast-track performance exceeded national standards without compromising cancer detection rates (~65% overall) or patient safety. The complication rate was very low, confirming the safety of the TP approach. Patient-reported outcomes indicate that although the procedure can cause discomfort, it was generally well tolerated given the advantages of avoiding general anesthesia and hospitalization. Our single-center experience supports broader use of clinic-based LATP pathways to speed diagnosis and improve patient convenience, with general anesthesia reserved for the minority who need it.

## Introduction

Prostate cancer is the most common solid malignancy among men in many countries. In the United Kingdom, over 50,000 new cases are diagnosed each year, and approximately 12,000 men die of the disease annually [1]. Early and accurate diagnosis is critical for optimal outcomes, and healthcare systems have focused on streamlining diagnostic pathways. Traditionally, prostate biopsies have been performed via the transrectal ultrasound-guided (TRUS) approach, often in an operating theater under sedation or general anesthesia. While effective for sampling, the TRUS route carries a risk of infectious complications due to needle passage through rectal flora. Reported post-TRUS biopsy infection rates range from 1% to 3%, with sepsis occurring in roughly 1-2% of patients even with prophylactic antibiotics [2]. These infections can be serious, sometimes requiring hospitalization, and there is growing concern about antibiotic resistance resulting from widespread prophylactic use in TRUS biopsy protocols.

In recent years, the transperineal (TP) approach to prostate biopsy has gained international favor as a safer and potentially more accurate alternative. The TP route involves sampling the prostate through the perineal skin, thereby virtually eliminating direct fecal contamination. Mounting evidence indicates that TP biopsy significantly reduces infection risk compared with TRUS. For example, the landmark PREVENT trial, a multicenter randomized study, reported zero cases of post-biopsy infection in an office-based TP arm (performed under local anesthesia without antibiotics), compared with a 1.4% sepsis rate in the transrectal arm (despite targeted antibiotic prophylaxis) [2]. Importantly, the PREVENT trial found no compromise in cancer detection: both approaches achieved equivalent rates of clinically significant prostate cancer detection [2]. Other studies and meta-analyses have likewise confirmed that TP and transrectal techniques have comparable overall detection rates, with no significant difference in identifying clinically significant cancer [3]. The TP approach may also sample anterior regions of the prostate more effectively, potentially improving detection of anteriorly located tumors [3]. These advantages are achieved without imposing a substantial additional burden on patients.

Patient tolerability of TP biopsy under local anesthesia has been shown to be acceptable in most cases. Many patients report that the experience is worthwhile given the benefit of avoiding a hospital admission or general anesthesia [4]. Proper local anesthetic technique (e.g., periprostatic nerve block and adequate infiltration) and appropriate counseling can make the procedure well tolerated by the majority of men [4]. Indeed, one recent series reported that no patients experienced uncontrolled pain and none developed sepsis when undergoing outpatient TP biopsy under local anesthesia [4]. Reflecting these benefits, clinical practice guidelines in the UK have evolved to endorse the TP approach in the outpatient setting. The National Institute for Health and Care Excellence (NICE) issued Diagnostics Guidance DG54 in June 2023, which concluded that TP prostate biopsy under local anesthesia is an effective option with significantly lower infection risk than TRUS, without compromising cancer detection [5]. Around the same time, the “Getting It Right First Time” (GIRFT) Urology program, an NHS improvement initiative, released updated best-practice recommendations (April 2024) for suspected prostate cancer pathways. These guidelines explicitly recommend offering clinic-based TP prostate biopsy under local anesthesia (LATP) as the default technique for prostate tissue diagnosis [6]. In addition, GIRFT and NHS England established an ambitious Faster Diagnosis Standard (FDS) for cancer pathways: by October 2023, at least 75% of patients referred with suspected cancer should receive a diagnosis (or have cancer ruled out) within 28 days of referral, with the target increasing to 80% by 2026 [6]. For prostate cancer, this has driven the development of “one-stop” or rapid diagnostic pathways, aiming for turnaround times as short as 14 days from referral to outcome.

A district general hospital in South West London implemented a new clinic-based LATP biopsy pathway in 2024, in line with the national guidance outlined above. This represented a significant change from the institution’s prior standard of care, in which most prostate biopsies were performed under sedation in the operating theater, often with attendant scheduling delays. We conducted a retrospective evaluation of the first six months of the LATP pathway to assess its performance and outcomes. Our objectives were to measure diagnostic timeline targets, diagnostic yield, compliance, complication rates, and patient-reported experience. We hypothesized that the LATP pathway would substantially shorten time to diagnosis and reduce infectious complications compared with historical practice, without compromising cancer detection, and that patient satisfaction would remain high given the convenience and safety benefits of avoiding general anesthesia.

## Materials and methods

Study design and setting

This was a retrospective observational audit conducted at a district general hospital in South West London, UK. We reviewed all adult men who underwent an outpatient LATP prostate biopsy as part of the new “one-stop” prostate cancer diagnostic pathway over a six-month period (May 1, 2024 to November 30, 2024). This timeframe corresponds to the initial rollout of the LATP pathway at our institution.

As this project was a clinical audit and service evaluation of an implemented pathway, formal research ethics committee approval was not required. All data were collected and managed in accordance with institutional clinical audit governance standards. Patient consent for the procedure and the follow-up survey was obtained as part of routine care.

Patient cohort

All men referred via the urgent suspected prostate cancer referral pathway (two-week-wait referrals) who underwent a TP prostate biopsy under local anesthesia in the clinic were included. Referral indications typically included elevated prostate-specific antigen (PSA) and/or an abnormal digital rectal examination suspicious for prostate cancer.

Patients who required upfront general anesthesia for prostate biopsy (e.g., those unable to tolerate the procedure under local anesthesia or with contraindications to awake biopsy) were managed under the prior pathway (theater-based biopsy) and excluded from this audit. We also excluded patients who did not complete the diagnostic pathway (e.g., biopsy recommended but deferred or not performed due to a change in clinical plan).

In total, 115 consecutive patients met the inclusion criteria and formed the study cohort. An additional 35 patients were excluded due to the requirement for general anesthesia.

LATP pathway protocol

The pathway was structured to achieve diagnosis within the 28-day FDS. After urgent referral, patients attended a one-stop urology clinic for clinical evaluation, including history, physical examination with DRE, and blood tests (PSA if not already completed by the GP). A multiparametric MRI of the prostate was arranged promptly, typically within seven days of referral.

If MRI showed suspicious lesions (PIRADS 3-5), or if clinical suspicion remained despite a non-targetable MRI, patients were scheduled for a clinic-based LATP biopsy. Biopsies were performed in a dedicated outpatient procedure room by a consultant urologist using an ultrasound-guided freehand TP technique. Targeting was performed cognitively using Koelis software. A stabilizing stepper or grid was not routinely used; instead, a PrecisionPoint needle guide attached to the ultrasound probe facilitated sampling.

Local anesthesia consisted of ~10-20 mL of 1% lidocaine infiltrated into the perineal skin and along anticipated needle trajectories, with periprostatic nerve block as needed. The biopsy protocol included targeted cores (up to 4 per PIRADS 3-5 lesion) combined with a systematic extended-sextant 12-core sampling, unless modified by clinical judgment. Patients were observed briefly post-procedure and discharged the same day with written advice and contact information.

Pre-biopsy safety checks were performed on the day of the procedure. Vital signs (heart rate, blood pressure, and oxygen saturation) were recorded, and urinalysis was conducted if a recent urine culture was unavailable. The biopsy proceeded only if parameters were within normal limits and no evidence of active infection was present (e.g., negative urine dipstick or adequately treated UTI).

Unlike some published protocols, our institution used antibiotic prophylaxis: all patients received a prophylactic intramuscular gentamicin injection prior to biopsy. This decision was made locally to provide additional safety during early implementation, despite evidence that infection rates are low even without antibiotics in TP biopsy [2].

Biopsy samples were processed by specialist uropathologists, and results were reviewed at the weekly multidisciplinary team (MDT) meeting. The definitive diagnostic outcome was defined as the communication of biopsy results (cancer or benign findings) to the patient along with an agreed management plan. In most cases, this occurred at a follow-up clinic visit or telephone call shortly after MDT discussion.

For patients with positive biopsies, key pathological details (Gleason grade group, number of positive cores, and tumor extent) were recorded for context, though detailed tumor characterization was beyond the scope of this pathway-focused study.

Data collection

We utilized the hospital’s electronic patient record system and cancer tracking database to identify all patients who had a local anesthetic TP prostate biopsy coded during the audit period (clinic coding label “LA TPPB”). From these records, we extracted relevant data for each patient, including age, dates of key pathway milestones (referral, MRI, biopsy, MDT review, and definitive diagnosis communication), biopsy results (presence or absence of prostate cancer and Gleason score if positive), and any complications or unplanned healthcare encounters within 30 days post-biopsy. Complication data were obtained by reviewing clinic notes, hospital admissions, emergency department records, and primary care correspondence for the 30 days following the biopsy. This was actively done by searching the patients’ records, where we specifically noted any episodes of fever/sepsis, urinary retention, bleeding, vasovagal events, or other adverse outcomes. For a longer-term context, we cross-checked subsequent clinic letters for any delayed issues related to the biopsy.

Patient experience data were collected via a post-procedure survey. Several weeks after the biopsy, patients were contacted by telephone to obtain verbal consent and then emailed a questionnaire about their experience. The survey included the following items: (1) overall satisfaction: “How satisfied were you overall with the prostate biopsy experience?” (scale 0-10, where 0 = not at all satisfied and 10 = extremely satisfied); (2) a follow-up to Q1 asking for any specific comments explaining the chosen satisfaction score (open-ended free-text response); (3) pain rating: “How painful did you find the procedure?” (numeric scale 0-10, where 0 = no pain and 10 = worst pain imaginable); (4) tolerability rating: “How well did you think you tolerated the procedure?” (numeric scale 0-10, where 0 = not difficult at all to tolerate and 10 = extremely difficult to tolerate); (5) expectations regarding discomfort: “How did the procedure compare to your expectations in terms of pain and discomfort?” (multiple choice: better than expected / as expected / worse than expected); (6) expectations regarding duration: “How did you find the overall time spent in hospital compared to your expectations?” (multiple choice: shorter than expected / as expected / longer than expected); (7) future preference: “If you need another prostate biopsy in the future, would you prefer to have it done under local anesthesia (same way as this time)?” (options: Yes - would choose local again / Not sure - undecided / No - would prefer general anesthesia or sedation next time); (8) post-procedural complications: “Did you experience any post-procedure complications or side effects in the days following the biopsy?” (Yes/No; if yes, respondents were prompted to specify the complication); (9) a follow-up to Q8: “If yes, was the complication minor or severe?” (options: Minor - managed at home / Severe - required medical attention); (10) clinician communication - reason: “Rate the clinician on explaining the reason for the biopsy” (Likert scale: very well / adequately / not well, etc.); (11) clinician communication - clarity: “Rate the clinician on explaining the procedure in a clear and easy-to-understand way” (Likert scale as above); (12) clinician communication - questions: “Rate the clinician on answering your questions” (Likert scale as above); and (13) additional feedback: “Do you have any other comments or feedback about your experience with the local anesthetic prostate biopsy procedure?” (open-ended free-text).

Outcome measures and analysis

The primary outcomes were diagnostic timeline metrics (referral-to-biopsy time and referral-to-definitive-diagnosis time) and the proportion of patients meeting the 28-day FDS. We calculated the mean, median, and range for each time interval. The percentage of patients diagnosed within 28 days was determined and compared with the national FDS target (77% at the time) [6]. Given the audit nature of the study, formal hypothesis testing was limited. Ninety-five percent CIs were constructed for major proportions (such as the FDS compliance rate) to assess significance against targets. A one-sample test for proportion was used to evaluate whether the observed FDS achievement significantly exceeded 77%. Secondary outcomes included the cancer detection rate on biopsy (overall and clinically significant cancer yield) and 30-day post-biopsy complication rates. Patient-reported outcome measures from the survey were summarized as mean ± SD for pain, tolerability, and satisfaction scores, and as the distribution of responses for the anesthesia preference question. All statistical analyses were descriptive; no comparative subgroup analyses were performed. Results from our pathway are contextualized by comparison with national standards and relevant findings in the literature where appropriate.

## Results

Diagnostic timeliness and yield

The LATP pathway achieved notably short diagnostic times. The mean interval from urgent GP referral to biopsy was 9.96 ± 2.5 days (median, 10 days). The mean time from biopsy to definitive diagnosis (histopathology result with MDT review) was 4.76 ± 1.2 days (median, 5 days). Consequently, the overall mean referral-to-diagnosis time was 14.5 ± 3.1 days (median, 14 days), substantially shorter than historical waits of several weeks under the theater-based system. More than 60% of patients completed the entire diagnostic process in approximately two weeks. Table 1 summarizes the timeline metrics and performance relative to national standards.

**Table 1:** Diagnostic pathway timeline and FDS performance Times were calculated from receipt of urgent referral to biopsy and from biopsy to MDT-confirmed diagnosis. At the time of the study, the UK 28-day FDS benchmark was 77%; our pathway achieved 85.4%, which was significantly above the target (χ²(1) = 4.38, p = 0.036). FDS, Faster Diagnosis Standard; GG, grade group; MDT, multidisciplinary team

Metric	Mean	SD	Median	Range	Percentage	n	N	Test statistic	p-value	95% CI
Referral-to-biopsy time (days)	9.96	2.5	10	3-21	N/A	N/A	N/A	N/A	N/A	N/A
Biopsy-to-diagnosis time (days)	4.76	1.2	5	2-10	N/A	N/A	N/A	N/A	N/A	N/A
Total referral-to-diagnosis time (days)	14.47	3.1	14	7-30	N/A	N/A	N/A	N/A	N/A	N/A
Met the 28-day diagnosis standard	N/A	N/A	N/A	N/A	85.40%	98	115	χ²(1) = 4.38	0.036	79.0-91.9%
Cancer detected on biopsy	N/A	N/A	N/A	N/A	65.20%	75	115	N/A	N/A	N/A
Clinically significant cancer (GG ≥2)	N/A	N/A	N/A	N/A	76.00%	57	75	N/A	N/A	N/A

As shown in Table 1, the mean total time from referral to diagnosis was ~14 days, well under the 28-day standard. The distribution of total times (not shown) was slightly right-skewed: most patients were diagnosed within 10-20 days, with a small tail of outliers approaching 28-30 days. Overall, 98 of 115 patients (85.4%) received a definitive diagnosis within 28 days of referral, comfortably exceeding the national performance threshold of 77% [6]. The 95% CI for our on-time diagnosis rate was 79.0-91.9%, entirely above 77%, confirming statistically significant outperformance of the target (p ≈ 0.03). Only 17 patients (14.6%) missed the 28-day benchmark, usually by a narrow margin of a few days. Review of these cases revealed minor, case-specific delays (e.g., patient rescheduling conflicts, need for repeat imaging, or scheduling around public holidays) rather than systemic bottlenecks. No consistent issue was identified that delayed the pathway. These results indicate that the new LATP clinic pathway can reliably deliver rapid diagnosis for most patients.

In terms of diagnostic yield, 75 of 115 biopsies were positive for adenocarcinoma, giving an overall cancer detection rate of 65.2%. This aligns with expectations for an MRI-targeted biopsy cohort (all patients had PIRADS 3-5 lesions or high clinical suspicion) and suggests that moving to TP biopsy did not compromise cancer detection. Published studies report that cancer detection with TP approaches is at least equivalent to transrectal biopsy and may be superior for anteriorly located tumors that TRUS biopsy can miss [2,3]. In our series, 57 of the 75 cancers (76%) were Gleason Grade Group ≥2, i.e., clinically significant prostate cancer requiring treatment or active surveillance, while the remaining 18 cancers (24%) were Grade Group 1 (Gleason 3+3=6, considered low-risk disease). Thus, most detected cancers were clinically significant. All cancer cases were discussed in MDT and managed according to risk, ranging from active surveillance to radical therapy. Patients with negative biopsies or only low-risk cancer were returned to routine monitoring. Importantly, no MRI-suspicious lesion yielded a missed cancer due to the biopsy approach; specifically, no anterior zone tumors went undetected, indicating that the TP technique adequately sampled the targets.

Safety and complications

The clinic-based LATP procedure was very well tolerated, with minimal complications observed. Notably, there were no cases of post-biopsy urinary retention. A total of four patients (3.5% of 115) experienced complications within 30 days of biopsy (Table 2). The overall complication rate of ~3.5% included only minor and moderate events, with no major adverse events. Two patients (1.7%) developed urosepsis post-biopsy. Both had multiple medical comorbidities, presented with fever within a few days of the procedure, and were hospitalized for intravenous antibiotics. In both cases, *Escherichia coli *was identified, and patients were treated with a five-day course of ceftazidime plus a single dose of gentamicin, in line with trust guidelines. Both recovered fully within 48 hours with no lasting sequelae. Another two patients (1.7%) experienced vasovagal reactions during or immediately after the biopsy, presenting with transient hypotension and light-headedness. These were managed conservatively in the clinic (observation, fluids, and rest), and both recovered within minutes with no further issues. No other adverse events were observed during the 30-day post-biopsy period; specifically, there were no cases of significant bleeding requiring intervention, thromboembolic events, procedure-related deaths, intensive care admissions, or long-term complications. The absence of severe sepsis or other major complications supports the safety of the LATP approach in the outpatient setting.

**Table 2:** Patient-reported outcomes and 30-day complication rates (LATP pathway) Patient-reported outcome data are from the post-biopsy survey (48 respondents, ≈42% of the cohort). The tolerability scale was inversely scored (lower numbers = more tolerable/easier to endure). “Would choose LATP again” reflects patient preference if a future biopsy were needed (respondents who answered “No” would prefer general anesthesia or sedation next time). Complication rates refer to events within 30 days post-biopsy; percentages are based on the full cohort (N = 115), and no patient experienced more than one complication. GA, general anesthesia; LATP, transperineal prostate biopsy under local anesthesia

Outcome measure	Mean	SD	Percentage	n	N
Patient satisfaction (0-10)	8.2	1.4	N/A	N/A	48
Pain during biopsy (0-10)	6.2	2.3	N/A	N/A	48
Procedure tolerability (0-10, higher = more difficult)	3.2	2.8	N/A	N/A	48
Would choose LATP again - Yes	N/A	N/A	68.0%	33	48
Would choose LATP again - Unsure	N/A	N/A	20.0%	9	48
Would choose LATP again - No (prefer GA/sedation)	N/A	N/A	12.0%	6	48
Any complication (within 30 days)	N/A	N/A	3.5%	4	115
Urosepsis requiring admission	N/A	N/A	1.7%	2	115
Vasovagal episode (no admission)	N/A	N/A	1.7%	2	115
Other complications	N/A	N/A	0.0%	0	115

From a patient-experience perspective, feedback was generally positive. Forty-eight patients returned the post-biopsy survey (~42% response rate), though this should be considered in interpretation, given the potential for response bias. Table 2 summarizes key patient-reported outcome metrics. The mean satisfaction score with the overall diagnostic process was 8.2 ± 1.4 out of 10, indicating a high level of satisfaction on average. Patients did report experiencing pain during the biopsy: the mean pain score was 6.2 ± 2.3 out of 10 (where 10 = “worst pain imaginable”). Pain scores varied widely; about 15% of respondents rated pain as ≥8 (severe pain), roughly 30% rated pain as ≤4 (mild discomfort), and the remainder reported moderate pain in the 5-7 range.

The mean tolerability rating was 3.2 ± 2.8 on a 0-10 scale (where 10 = “extremely difficult to tolerate”). Thus, on average, patients placed the experience on the easier end of the tolerability spectrum, consistent with the notion that while the biopsy caused some discomfort, it was manageable for most. Notably, 40 of 48 respondents (≈83%) rated tolerability ≤5 (relatively easy to tolerate). These findings are consistent with prior studies reporting that TP biopsy under local anesthesia, though sometimes more painful than TRUS biopsy, is generally well tolerated with appropriate analgesia [7].

When asked if they would opt for the same biopsy method again under local anesthesia, the majority responded positively: 68% of surveyed patients said they would choose LATP again if another biopsy were needed, 20% were unsure, and only 12% said they would prefer an alternative such as sedation or general anesthesia. This indicates that about two-thirds of patients were sufficiently satisfied to repeat the procedure under local anesthesia, while roughly one in eight would prefer deeper anesthesia or a different approach, likely reflecting those who experienced higher pain or anxiety. The remaining ~20% were ambivalent.

Free-text comments provided additional context. Many patients expressed appreciation for the speed of diagnosis (“I was relieved to get my results so quickly”) and the convenience of avoiding a hospital stay. A few described the biopsy as painful or anxiety-provoking but often added that receiving prompt results “made it worthwhile.” Overall, the patient-reported outcomes suggest that with appropriate analgesia, patient education, and support during the procedure, an outpatient LATP biopsy is acceptable to most patients, even if not entirely pain-free.

## Discussion

This study provides a real-world evaluation of a newly implemented clinic-based LATP prostate biopsy pathway in an NHS setting. The results demonstrate that the pathway achieved its primary goals: meeting national diagnostic timelines while maintaining acceptable complication rates, thereby offering a practical alternative to sedation-based biopsies. Below, we discuss the implications of these findings in the context of existing literature and practice.

Diagnostic speed and pathway performance

Our LATP pathway enabled an average referral-to-diagnosis time of approximately 14 days, with more than 85% of patients receiving a definitive diagnosis within 28 days of referral. The pathway’s performance exceeded the current NHS FDS (75-77% within 28 days) and approached the future target of 80%. A large majority of patients were diagnosed well within 28 days, and many within about two weeks. Achieving a two-week diagnostic pathway for prostate cancer has been cited as an ideal scenario in pilot programs [6], and our results demonstrate that this is feasible with efficient process organization. This not only meets national performance targets but also likely provides clinical benefits: earlier diagnoses can translate into earlier treatment for those with cancer or more rapid reassurance for those without cancer, thereby reducing anxiety and potentially improving outcomes.

Other centers in the UK that have adopted similar approaches are likewise reporting substantial improvements in diagnostic timelines. For example, some institutions have piloted same-day MRI and biopsy clinics, reporting median pathway durations under three weeks while improving FDS compliance (these data have appeared in conference abstracts and regional audits). One downstream consequence of faster diagnosis is that patients diagnosed with cancer can be discussed at MDT and commence therapy sooner, while those without cancer spend less time in uncertainty and can be discharged or monitored with reduced anxiety. Although our study did not specifically measure psychological or long-term clinical outcomes, the acceleration of the diagnostic process is inherently patient-centered and aligns with the NHS's aim of faster diagnosis without compromising care.

It should be noted that our study did not include a direct comparison with the prior pathway, as we did not collect systematic data on referral-to-diagnosis times when biopsies were performed under the old system. However, national data around the time of our transition showed that many trusts were struggling to meet the 75% 28-day FDS target for prostate cancer [6]. Future work could formally compare pre-implementation and post-implementation performance to quantify the gains provided by the LATP model, but even without such analysis, our results clearly indicate that rapid prostate cancer diagnosis is achievable in practice through adoption of the recommended pathway changes.

Safety profile

The safety results from our audit strongly support the superiority of the LATP approach in reducing infectious complications. We observed a urosepsis rate of 1.7% (2/115 patients); importantly, all infections were manageable, and none required intensive care or resulted in prolonged morbidity. This rate is as low as, or lower than, typical rates reported for TRUS biopsy, which have historically been around 1-3% even with antibiotic prophylaxis [2]. Large comparative studies have likewise demonstrated significantly lower post-biopsy sepsis rates with TP approaches compared to transrectal [8].

It is worth noting that our patients did receive prophylactic antibiotics (gentamicin), despite evidence that TP biopsies may not require them [2]. It is possible that our sepsis rate could have been even lower had we adopted a completely antibiotic-free approach, as some centers have done; indeed, minimizing prophylaxis is desirable given the global rise in antibiotic-resistant infections [9]. Nonetheless, even with prophylaxis, transrectal biopsies carry a non-negligible infection risk, whereas our data show that a properly implemented TP pathway can virtually eliminate severe post-biopsy infections.

Beyond infections, the absence of serious hemorrhagic complications or urinary retention in our series is also reassuring. TP biopsy, by avoiding traversal of the rectal wall, eliminates the risk of transrectal bleeding, and our use of small-gauge needles and careful technique likely contributed to the absence of significant bleeding events.

It is also notable that none of our patients required hospital admission for reasons other than the two infection cases. This contrasts with historical TRUS practice, where post-biopsy sepsis was a leading cause of urological emergency admissions. The near-elimination of that problem in our cohort underscores a major system-level benefit of the LATP pathway. For example, the National Prostate Cancer Audit in England reported a marked decline in post-biopsy hospitalizations for sepsis following widespread adoption of TP biopsy [10]. This improvement is further reinforced by global surveillance studies showing rising antimicrobial resistance in post-TRUS infections, strengthening the rationale for TP techniques in mitigating these risks [9].

Patient experience and acceptability

Our preliminary findings suggest that performing prostate biopsies in the clinic under local anesthesia results in moderate tolerability. The patient-reported outcomes are consistent with published studies indicating that while LATP can cause moderate pain, the difference in pain compared with TRUS under local anesthesia is generally modest and short-lived [7]. Moreover, any increased discomfort with the TP approach can be mitigated through techniques such as improved local anesthesia (e.g., periprostatic block or more extensive infiltration), use of oral analgesics or anxiolytics, and appropriate patient coaching. In our series, although some patients experienced significant pain, the overall tolerability scores were low (indicating good tolerance), even within our limited response set. This likely reflects effective local anesthesia administration and clear communication of expectations.

Most importantly, a large majority of patients (over two-thirds) indicated that they would choose the same method again. This finding mirrors reports in the literature, where 85-94% of patients state they would undergo a TP biopsy under local anesthesia again if required [11,12]. While a subset of patients would prefer sedation or general anesthesia, for most individuals, the trade-off of some discomfort in exchange for a quick, outpatient procedure is acceptable. For the minority with significant anxiety or low pain tolerance, an individualized approach, such as offering sedation, is appropriate, but our data suggest that routine use of general anesthesia is unnecessary in most cases.

Resource implications and cost-effectiveness

An often-cited advantage of moving prostate biopsies out of the operating theater is the saving of healthcare resources and costs. Although a full health economic analysis was beyond the scope of our audit, some general observations can be made. By avoiding general anesthesia and theater utilization, our clinic-based approach freed up operating theater slots for other surgical cases and eliminated the need for anesthetic staff and recovery beds for this diagnostic procedure. The literature also supports significant cost savings with local anesthetic TP biopsy compared with general anesthesia. For example, a recent cost-effectiveness analysis by Roberts et al. (2025) showed that performing TP biopsies under local anesthesia is cost-saving from a health system perspective, largely by avoiding theater costs and enabling faster patient throughput, with no compromise in diagnostic yield [13].

Our experience mirrors this: we were able to perform more biopsies per week in the outpatient setting than was possible when constrained by theater time, and none of our patients required hospital admission post-biopsy except the two with infections (a rate that, as noted, is lower than typical post-TRUS admission rates). These efficiency gains and cost savings further strengthen the case for widespread adoption of LATP pathways.

Limitations

This audit has several important limitations. It was a single-center, non-randomized study without a contemporaneous control group. Comparisons relied on historic or published data, which, while informative, are less robust than direct head-to-head analyses. The patient survey had a modest response rate (42%), introducing the possibility of response bias, as patients with particularly strong opinions or experiences may have been more likely to reply. A key reason for the low response rate was that each patient had to be contacted individually by phone to provide consent for the survey; many did not answer, and because this was a retrospective study, the survey option had not been mentioned at the time of biopsy.

There may also have been under-detection of minor complications such as hematospermia or mild urinary tract infections, since data collection relied on patients presenting to the hospital. In addition, follow-up was relatively short-term (30 days for complications and survey completion within a few months). Longer-term outcomes, such as missed or delayed cancer diagnoses, were not assessed, although the combined use of MRI and systematic sampling makes it unlikely that clinically significant cancers were overlooked. Finally, a learning curve effect must be acknowledged: as operators gain more experience with the LATP technique, patient comfort may improve further and procedure times may shorten, whereas our results include cases performed at the start of that curve.

Future directions

Our audit represents one of the first reports from a UK district general hospital setting following the 2023-24 guideline changes. The positive outcomes provide practical validation of the recommendations by NICE and GIRFT to adopt TP biopsy as the standard of care. We intend to continue monitoring pathway performance and to introduce incremental refinements to improve patient comfort (such as offering nitrous oxide). A formal comparison of pre- and post-implementation data would help quantify improvements in diagnostic timelines and complication rates.

As new biopsy devices and techniques emerge, such as robotic assistance or specialized freehand needle guides like CamPROBE, which has shown feasibility for in-clinic use [14], we will evaluate their potential benefits in our setting. Current evidence suggests that different freehand TP approaches have comparable efficacy [15], so our focus will remain on optimizing workflow and patient experience.

In summary, our experience demonstrates that a rapid, clinic-based LATP prostate biopsy pathway is both feasible and effective. It achieves timely diagnoses with a favorable safety profile, and most patients accept the trade-off of some additional discomfort in exchange for avoiding general anesthesia and hospitalization. Our findings should encourage other institutions to adopt this approach, as the benefits for patients and the healthcare system are considerable. Large multi-institutional studies internationally have also reported low complication rates and efficient cancer detection with outpatient TP biopsy programs [16]. This paradigm shift is increasingly reflected in international guidelines, with both the European Association of Urology and the American Urological Association highlighting TP biopsy as the preferred approach to reduce post-biopsy infections [17,18]. For the minority of patients unable to tolerate an awake procedure, individualized options such as targeted sedation or general anesthesia remain available, ensuring no patient is left behind.

## Conclusions

Implementation of an LATP biopsy pathway allowed us to exceed NHS diagnostic timeline standards while maintaining patient safety. Although patient feedback was limited, responses indicated generally positive satisfaction and moderate tolerability. This provides promising proof of concept for LATP as a feasible alternative to sedation-based prostate biopsy. Future prospective studies should incorporate direct comparisons between LATP and traditional pathways, include control groups, and assess long-term outcomes and cost-effectiveness.
